# The Influence of Postmenstrual Age and Neurological Impairments on the Modified Pain Assessment Tool Score in Infants Admitted to Neonatal Intensive Care: A Retrospective Medical Record Review

**DOI:** 10.1002/pne2.12133

**Published:** 2025-01-20

**Authors:** Jamie Murray, Jeremy Lam, Jeewan Jyoti, Kaye Spence, Himanshu Popat, Emre Ilhan

**Affiliations:** ^1^ Department of Health Sciences, Faculty of Medicine, Health and Human Sciences Macquarie University Sydney Australia; ^2^ Grace Centre for Newborn Intensive Care Children's Hospital at Westmead Sydney Australia; ^3^ Western Sydney University Sydney Australia; ^4^ NHMRC Clinical Trial Centre University of Sydney Sydney Australia

**Keywords:** infant pain, neonatal intensive care unit, neonatal pain, pain assessment

## Abstract

Repetitive and prolonged experience of pain by infants in neonatal intensive care units (NICUs) may adversely affect growth and alter pain responses. The degree of infant prematurity and/or presence of neurological impairment (NI) may impact an infant's ability to behaviorally respond to pain. This study aimed to determine whether the scores on the mPAT, a widely used pain assessment tool, is impacted by postmenstrual age (PMA) at assessment, irrespective of neurological impairment. Data from medical records were collected on infants admitted to the NICU who underwent a pain assessment with the modified Pain Assessment Tool (mPAT) between March 2019 and September 2021. Total mPAT, behavioral, and physiological pain scores were independently analyzed using logistic regression to detect differences based on PMA categories (< 33 weeks, 33–36 weeks, ≥ 37 weeks) and presence of NI. Significant differences were indicated when *p* < 0.05. Of 204 infants sampled, 62% were male, and 71% were born at term‐age (i.e., ≥ 37 wks). Thirty‐six (18%) infants had a queried or confirmed NI and 28 (14%) infants were postsurgical. Logistic regression analysis showed that neither PMA nor presence of NI predicted pain for total mPAT scores (*χ* ^2^ (3) = 3.9, *p* > 0.05) or physiological scores (*χ* ^2^ (3) = 2.7, *p* > 0.05). Higher behavioral scores were 3.7 times (OR 0.27, 95% CI 0.10–0.77, *p* = 0.01) more likely in extremely‐to‐very preterm (< 33 weeks) infants when compared to term (≥ 37 weeks) infants. The mPAT may be suitable for clinicians to utilize when assessing infants in NICUs regardless of PMA or NI status. The higher behavioral responses in younger infants require further investigation in a future prospective study.

## 1 Introduction

Advances in the neonatal intensive care unit (NICU) have led to neonates (infants less than 28 days old) and infants (less than 1 year old) surviving beyond hospitalization but also experiencing repeated painful procedures [1]. Neonates and infants can experience pain acutely following procedures such as heel lancing and nonacutely following major surgery [2]. The consequences of repeated painful procedures in the NICU include an increased risk of the development of prolonged pain [3], neurodevelopmental impairments [3], and alterations in pain sensitivity [1, 3], which highlight the importance of detecting pain accurately to minimize the negative effects of repeated pain later in life [1].

Pain assessment tools exist that measure various behavioral (e.g., muscular tone and facial expression) [4] and/or physiological indicators (e.g., heart rate and oxygen saturation) [4, 5], and/or contextual cues (e.g., gestational age), directing clinicians to appropriate pain reduction protocols [6]. Given the multidimensional nature of pain [7], such tools must incorporate behavioral and nonbehavioral indicators of pain [8], particularly as there is the potential for inappropriate pain management as a result of an underdetection of pain in preterm and neurologically impaired infants [8, 9, 10].

The Pain Assessment Tool (PAT) is a multidimensional pain assessment tool [11] that is currently used throughout NICUs in Australia and New Zealand. Recently, it has been modified to include additional descriptors to aid scoring, resulting in the modified PAT (mPAT) [12]. While the PAT has demonstrated reasonable inter‐rater reliability (*intraclass correlation coefficient* = 0.85) and concurrent validity (*r* = 0.76) for the use in detecting pain following surgery [13], the mPAT has demonstrated reasonable inter‐rater reliability (*intraclass correlation coefficient* = 0.83 at baseline, 0.86 at heel lance) and good concurrent validity (*r* = 0.81 at baseline, *r* = 0.91 at heel lance) for detecting acute episodic pain [12], highlighting a need to determine its use in nonacute pain states. The psychometric properties of the PAT and mPAT are comparable to other neonatal and infant pain assessment tools for use in the context of postsurgical and acute episodic pain [11]. The mPAT utilizes behavioral and physiological parameters as well as a stand‐alone, contextual item of nurse's perception to determine whether a neonate or infant is experiencing pain. It is used for nonacute pain, which specifically refers to pain that is not associated with an acute, noxious event. The mPAT, which is scored out of a total of 20 and indicates more intense pain with higher scores, is used to help decision‐making about pain management.

Guidelines for the use of the mPAT in guiding pain management stipulate adaptations based on the baseline state of the neonate or infant. Muscle‐relaxed and sedated infants are scored out of 12 by assessing only physiological parameters [14], as they will not have the ability to respond behaviorally. The mPAT, however, does not consider other variables that may minimize the expression of behavioral indicators of pain such as prematurity [8, 9]. In fact, discriminatory facial expressions in response to noxious stimuli seem to emerge at approximately 33‐weeks' gestation [15]. Consequently, it can be surmised that extremely and very preterm infants may lack the necessary motor capacity to mount a behavioral response to pain that is sufficient to be detected by clinicians. The problem of underdetection is compounded in the critical care setting of the NICU, where infants are sedated and muscle‐relaxed, further reducing their capacity to respond behaviorally to pain [16]. However, relying solely on physiological indicators of pain as a measure of pain may be inadequate, particularly as physiological markers such as heart rate are shown to be less responsive to noxious stimuli the more an infant is exposed to painful procedures [17].

The *Échelle de Douleur et d'Inconfort du Nouveau‐né* (EDIN) scale is behavioral neonatal pain assessment tool used for assessing prolonged pain in critically ill neonates and infants. It has demonstrated good levels of inter‐rater reliability and construct validity [18]. However, a study by Ancora et al. [19] found that the EDIN scale was better able to detect pain when the tool specifically accounted for gestational age (time elapsed from the last menstrual period to delivery date) and postmenstrual age (PMA, time elapsed since birth plus gestational age). Thus, the modified version of the EDIN scale assigned extra points to infants who were younger due to their reduced ability to express pain behaviorally. While previous research has validated the mPAT for acute pain assessment in infants born ≥ 24‐weeks' gestation [12], the mPAT requires further validation to assess nonacute pain and whether it is reliable across gestational ages.

Validation of the mPAT for potentially neurologically impaired populations is required, particularly as this cohort is often excluded from neonatal pain research. The facial expressions of neonates who are at higher risk of neurological impairment may not be as pronounced compared to those with no or lower risk of impairment [10, 20]. A survey conducted by Stevens et al. [21] found clinicians tend to rely more on physiological indicators than behavioral indicators during the assessment of acute pain in infants who are at risk of neurological impairment, which suggests that behavioral indicators of pain in this cohort may not be reliable.

Therefore, there is an obvious gap in knowledge leading to uncertainty about the reliability of using the mPAT for pain assessment in preterm and infants at risk of neurological impairment. To address this gap, the aim of the present study is to determine whether the mPAT is influenced by postmenstrual age at the time of assessment or the risk of neurological impairment. Therefore, this study has two research questions:

1. Does postmenstrual age at the time of assessment influence the total score, and/or the behavioral and physiological scores of the mPAT?
2. Does the risk of neurological impairment influence the total score, and/or the behavioral and physiological scores of the mPAT?

## 2 Methods

### 2.1 Study Design

This study was a retrospective analysis of medical records of infants that were admitted into Grace Centre for Newborn Intensive Care at the Children's Hospital Westmead, Sydney, Australia. This Centre is a Level III specialized unit at a tertiary hospital providing care to critically ill neonates and infants with complex medical conditions such as serious cardiac and surgical disorders. This study was designed to align with the STROBE checklist for cross‐sectional studies [22], with any missing components being supplemented by the ISPOR retrospective database analysis checklist [23]. This project (LNR/18/SCHN/298) was approved by the Human Research Ethics Committee of the Sydney Children's Hospital Network.

### 2.2 Eligibility Criteria

This study included all infants in the NICU who underwent a pain assessment using the mPAT between March 2019 and September 2021. Infants who did not undergo a pain assessment or infants with neonatal abstinence syndrome from maternal drug use were excluded from the study.

### 2.3 Data Collection

Data for this study were collected from an electronic medical record system (PowerChart, Cerner). Due to difficulty in obtaining time dependent variables, only the initial mPAT assessments conducted in the NICU were extracted and subsequent assessments were not extracted. Collected variables included scores on each of the mPAT parameters (Table 1), birthweight, sex assigned at birth, gestational age at birth, PMA at the time of mPAT assessment, primary reason for admission, query or presence of neurological impairment (e.g., Spina bifida and encephalopathy), any surgical procedures performed prior to the mPAT assessment, administration of medications such as analgesia, sedatives, or muscle relaxants within 12‐h prior to the initial pain assessment, ventilation type, clinician type, and action taken in response to the scoring of the pain assessment.

**TABLE 1:** Modified pain assessment tool (mPAT).

		Points		
Dimension	Parameter	0	1	2
Behavioral	1. Posture/Tone	Normal Some flexion	Digits widespread Trunk rigid Limbs drawn out Shoulders raised off bed	Fists clenched Trunk guarded Limbs drawn to midline Head and shoulders resist posturing
	2. Cry	No	Consolable Can be settled	When disturbed Does not settle after handling Loud Whimpering Whining
	3. Sleep Pattern	Relaxed	Easily woken	Agitated or withdrawn Wakes with startle Restless Squirming No clear sleep/wake pattern Eye aversion ‘shut out’
	4. Expression	Relaxed Normal	Frown Shallow furrows Eyes lightly closed	Grimace Deep furrows Eyes tightly closed Pupils dilated
Physiological	5. Color	Pink well perfused	Occasionally mottled or pale	Pale/dusky/flushed Palmar sweating
	6. Respirations	Normal baseline rate	Tachypnoea—at rest	Apnea—at rest or with handling
	7. Heart Rate	Normal baseline rate	Tachycardia—at rest	Fluctuating—spontaneous or at rest
	8. Oxygen Saturation	Normal	Fleeting desaturation	Desaturation with or without handling
	9. Blood Pressure	Normal		Hypo/hypertension at rest
Clinician's Perception	10. Nurse's Perception	“No pain perceived”	“I think the baby is in pain only with handling”	“I think the baby is in pain”

*Note:* A summative score is made out of 20. Scores ≤ 5 indicate no pain. Scores > 5 indicate moderate pain presence and need for nonpharmacological pain relief, continue current management or medication weaning. Scores ≥ 10 indicates severe pain presence and need to consider increasing or administering analgesia. A summative score is made out of 20. Scores ≤ 5 indicate no pain. Scores > 5 indicate moderate pain presence and need for nonpharmacological pain relief, continue current management or medication weaning. Scores ≥ 10 indicates severe pain presence and need to consider increasing or administering analgesia.

### 2.4 The mPAT

The modified Pain Assessment Tool (mPAT) comprises 10 parameters, each with corresponding descriptors, as shown in Table 1. Each item is scored on a scale of 0 to 2 and the aggregate score out of 20 is used to guide appropriate pain management. The clinician conducting the assessment observes the neonate's face and whole body for 2 min and then gently touches their limbs to evaluate muscle tone and record the scores. Any external factors that the clinician thought may have had an impact on the score should also be noted as well as the action(s) taken by the clinician as a result of the assessment [24]. Infants who are under the influence of muscle relaxants (e.g., vecuronium) are scored using only the physiological and nurse's perception parameters meaning that their total maximum score is 12 and the score thresholds to indicate are adjusted accordingly. Meaning ≥ 3 for pain requiring comfort care, and ≥ 5 for pain indicating analgesic management [14, 24].

### 2.5 Data Analysis

To detect variations in total mPAT score, behavioral and physiological pain subscores, infants were categorized based on PMA at assessment and neurological impairment risk status. PMA categories were classified as [19] extremely‐to‐very preterm (< 33 weeks), moderate‐to‐late preterm (33‐36 weeks), and term (≥ 37 weeks). Neurological impairment risk status was dichotomized to presence or suspicion of neurological impairment and no presence of neurological impairment. Infants who had muscle relaxants (e.g., vecuronium) within 12‐h prior to the initial pain assessment were excluded from any analyses.

The collected data were analyzed using IBM SPSS Statistics for Windows version 29.0.2.0 (Armonk, NY; IBM Corp). Logistic regression was used to analyze the influence of PMA and neurological impairment status on mPAT total score, as well as total behavioral and physiological pain scores, with a *p* value of < 0.05 indicating statistical significance. Prior to analysis, mPAT scores were dichotomized to indicate pain (a score > 5) or no pain. Because a score of > 5 equates to 30% of the total score, to evaluate behavioral pain separately, we calculated 30% of the total score of the behavioral items (30% of 8) to determine that a score ≥ 2 indicated behavioral pain. Similarly, to evaluate physiological pain separately, we calculated 30% of the total score of the physiological items (30% of 10) to determine that a score ≥ 3 indicated physiological pain. We used Nagelkerke's *R* ^2^ test to assess goodness‐of‐fit for logistic regression [25]. Based on the planned analyses, and proportion of infants with the outcome of interest set at 0.10, a desired power of 0.90, and a Type I error rate of 0.05, the minimum sample size required was 170 (jStat, JavaScript Library, 2023).

## 3 Results

### 3.1 Sample Demographics

**FIGURE 1:**
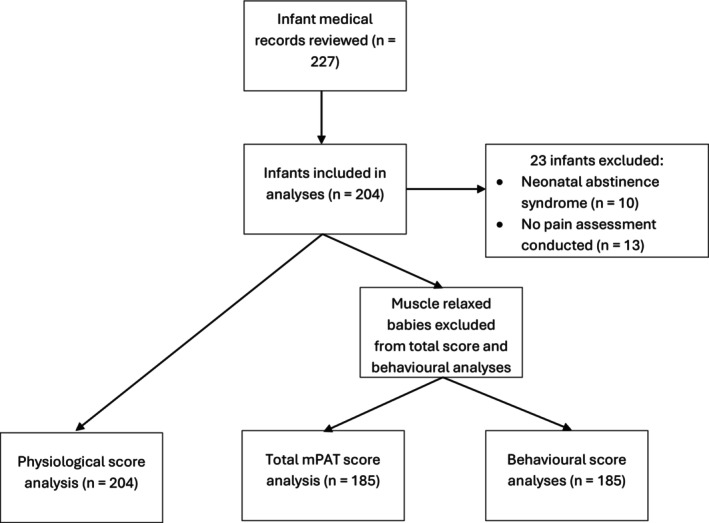
Participant flow through the analysis. mPAT indicates modified Pain Assessment Tool.

Of the 204 infants included in this study (Table 2, Figure 1), most were male (62%) and born at term‐age (≥ 37 wks, 71%). The remaining infants were moderate‐to‐late preterm (33–36 wks, 19%) and extremely‐to‐very preterm (< 33 wks, 24%). Ninety‐one infants (45%) were admitted for medical reasons, while 113 infants were admitted primarily for surgical interventions.

**TABLE 2:** Demographic and clinical characteristics at the time of pain assessment (*N* = 204).

Sex assigned at birth	
Male, *n* (%)	127 (62%)
Female, *n* (%)	77 (38%)
Gestational age (GA) at birth	
GA Mean ± SD, wks	35.5 ± 4.8
(< 33 wks) extremely‐to‐very preterm, *n* (%)	50 (24%)
(33–37 wks) moderate‐to‐late preterm, *n* (%)	38 (19%)
(37 ≥ wks) term, *n* (%)	116 (57%)
Postmenstrual age (PMA) at the time of pain assessment	
PMA Mean ± SD, wks	37.7 ± 3.7
(< 33 wks) extremely‐to‐very preterm, *n* (%)	22 (11%)
(33–37 wks) moderate‐to‐late preterm, *n* (%)	38 (19%)
(37 ≥ wks) term, *n* (%)	144 (70%)
Birth Weight	
Mean ± SD, gm	2529.8 ± 1100
Delivery Method	
Vaginal Delivery, *n* (%)	84 (41%)
Cesarean Section, *n* (%)	106 (52%)
Unknown, *n* (%)	14 (7%)
Ventilation type at time of pain assessment^a^	
% FiO_2_, Mean ± SD	27.6 ± 17.1
Spontaneous Ventilation—Room Air, *n* (%)	93 (46%)
Non‐Invasive Ventilation, *n* (%)	9 (4%)
Non‐Invasive Mechanical Ventilation, *n* (%)	45 (22%)
Invasive Mechanical Ventilation, *n* (%)	57 (28%)
Surgery	
Postsurgical patients at time of assessment, *n* (%)	28 (14%)
Presence/Query neurological condition (*n*)	36 (18%)
Medication at time of pain assessment^b^	
Analgesia, *n*	52
Sedative, *n*	16
Muscle relaxant, *n*	18
Reasons for admission	
Surgical, *n* (%)	113 (55%)
Medical, *n* (%)	91 (45%)

^a^ Noninvasive ventilation includes nasal prongs, high flow nasal prongs. Noninvasive Mechanical ventilation includes CPAP, BiPAP. Invasive Mechanical Ventilation includes endotracheal intubation, tracheostomy.

^b^ Medication at time of assessment (within ±12‐h) is not a mutually exclusive category.

**FIGURE 2:**
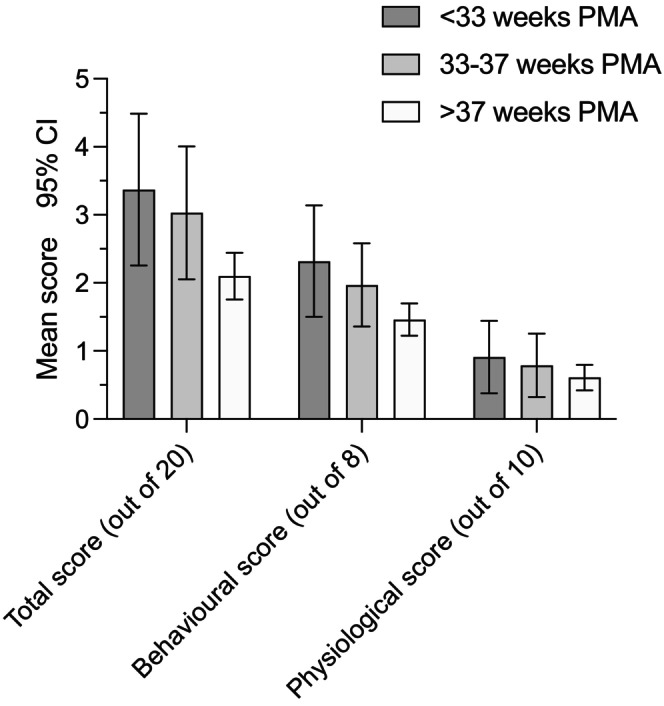
Mean total mPAT, behavioral, and physiological scores by postmenstrual age (PMA) group. Error bars represent upper and lower bounds of 95% confidence intervals.

Twenty‐four infants (13%) had a total mPAT score > 5 indicating the presence of pain, with three of these infants (2%) having a score ≥ 10 indicating severe pain. The average total pain score was 2.4 (SD: 2.2) (Figure 2). Nurses gave a score of 0 for nurse's perception of pain to 193 infants (90%).

Eighty‐two infants (44%) had a total behavioral score ≥ 2 indicating the presence of pain. Sixteen infants (8%) had a total physiological score ≥ 3 indicating pain presence. Thirty‐six (18%) infants had a queried or confirmed neurological impairment; however, only five had a total mPAT score ≥ 5. Only 6 (21%) of 28 postsurgical infants had pain presence indicated.

### 3.2 The Impact of Neurological Impairment and Postmenstrual Age at Assessment on Pain Scores

The analysis of the total mPAT score (Table 3) revealed that neither PMA nor the risk of neurological impairment were associated with pain presence, *χ* ^2^(3) = 3.9, *p* > 0.05. However, the logistic regression model using the behavioral pain score as the dependent variable was found to be significant, *χ* ^2^(3) = 9.16, *p* < 0.05. While the risk of neurological impairment did not independently predict the presence of behavioral pain (i.e., score of ≥ 2), the odds of pain in infants < 33 weeks PMA at assessment was 3.7 times more than infants ≥ 37 weeks PMA (odds ratio = 0.27, 95% CI 0.10 to 0.77, *p* = 0.01). No other pairwise comparisons were statistically significant (Table 3). In the physiological pain model, neither PMA nor the risk of neurological impairment was associated with the presence of physiological pain, *χ* ^2^(3) = 2.7, *p* > 0.05.

**TABLE 3:** Logistic regression analysis models (*N* = 204).

Variables	mPAT total pain					Behavioral pain					Physiological pain				
	B	Lower	OR	Upper	Sig.	B	Lower	OR	Upper	Sig.	B	Lower	OR	Upper	Sig.
Constant	−1.39		1			0.86		2.36			−2.47		1		
PMA 33–37^a^	0.02	0.25	1.02	4.12	0.98	−0.59	0.17	0.55	1.82	0.33	0.52	0.29	1.69	9.77	0.56
PMA ≥ 37^a^	−0.88	0.12	0.42	1.45	0.17	−1.29	0.1	0.27	0.77	0.01	−0.38	0.14	0.69	3.42	0.65
Neurological impairment^b^	0.25	0.43	1.29	3.86	0.65	−0.31	0.33	0.73	1.62	0.44	0.61	0.54	1.84	6.26	0.33

	*χ* ^2^	df	Sig.			*χ* ^2^	df	Sig.			*χ* ^2^	df	Sig.		
Omnibus tests of model coefficients	3.9	3	0.27			9.16	3	0.03			2.7	3	0.44		
Nagelkerke *R* Square			0.04					0.07					0.03		

Abbreviation: OR, odds ratio.

^a^ PMA < 33 is the reference category for PMA 33–37 and PMA ≥ 37 in each logistic regression model.

^b^ No neurological impairment is the reference category for neurological impairment in each logistic regression model.

## 4 Discussion

Our study demonstrated that PMA at assessment may not influence total mPAT or physiological pain scores. However, our findings demonstrated that extremely‐to‐very preterm infants (< 33 weeks PMA at assessment) were almost four times more likely to display behavioral indicators of pain than term‐age infants (≥ 37 weeks PMA). This study also demonstrated that the presence of neurological impairment did not influence the total mPAT score, and physiological or behavioral pain expression.

### 4.1 Influence of Neurological Impairment on the Expression of Pain

The results of this study indicate that the mPAT may be suitable for infants at risk of neurological impairment given that there was no significant difference in scores between those with and without risk of neurological impairment. A possible explanation can be found within the tool's scoring criteria for facial expression. Previous research has indicated that facial expression is a highly reliable, sensitive, and consistent behavioral indicator of pain [7, 10]; however, infants with neurological impairment may express fewer facial expressions than those without [20]. In a study by Gibbins et al. [26], it was established that brow bulge, eye squeeze and nasolabial furrow were the only facial expressions consistently indicating pain irrespective of neurological impairment. Thus, because these facial actions can be found within the mPAT, the mPAT may be suitable for assessing pain in infants at risk of neurological impairment. Alternatively, nurses' good clinical reasoning and understanding of contextual factors such as the impact of neurological impairment on pain expression may enable them to be more attuned to the behavioral expressions of infants with neurological impairment.

### 4.2 Influence of Post‐Menstrual Age on the Expression of Pain

The findings of our study contradict the current understanding that more premature infants are less likely to display behavioral indicators of pain due to immaturity of their nervous systems [15, 19, 26]. The higher behavioral scores in the extremely‐to‐very preterm infants compared with term infants in our study may be interpreted in two ways. Firstly, it is possible that the modifications made to the PAT provides criteria that allows nurses to adequately assess pain behaviorally regardless of an infant's PMA at assessment. This is consistent with O'Sullivan et al.'s [12] findings which demonstrated no significant difference in mean total mPAT scores between the three gestational age groups (≤ 28 wks, 28–37 wks, and ≥ 37 wks). The study also considered that the tool's criteria include precise descriptors such as “clenched fists” that preterm infants are capable of performing [12]. Thus, it can be hypothesized that the mPAT provides nurses more clarity as to what is considered normal or abnormal through the addition of descriptive criteria along the spectrum of behaviors that could indicate pain. These additional descriptors were not previously included in the PAT. It should be noted, however, that the study by O'Sullivan et al. [12] evaluated the mPAT following a painful procedure (i.e., acute pain states). Therefore, it is possible that responses to pain that is ongoing or prolonged, as was the case in our study, may be even more difficult to detect in extremely‐to‐very preterm infants. Indeed, there is an urgent need to develop tools for non‐acute pain that is appropriate across the developmental age spectrum [27].

The second interpretation could be explained by the ramifications of premature extrauterine exposure meeting specific behavioral criteria. The extrauterine life creates challenges for early development due to losing the antigravity, dark, quiet, and temperature‐regulated environment that they had while in utero [28]. Given that premature infants are often hypotonic and lack the independent or passive ability to flex against gravity, extension becomes an overpowering force [29, 30]. An “extended posture” meets the criteria for one point to be allocated behaviorally, despite the fact that preterm infants may not be able to functionally perform “some flexion” which is considered as normal in the mPAT descriptor for posture/tone. Similarly, “eyes tightly closed” and “no clear sleep/wake‐pattern” as descriptors for expression and sleep pattern, respectively, are criteria that could better be accounted for by nonpainful responses to the environment such as the stress related to exposure to light, noise, and frequent procedures/handling [31]. Indeed, a study conducted by Grunau et al. [32] found that preterm infants have a lower threshold and a more pronounced reflex response to touch compared with full‐term infants. Although nurses may consider extrauterine effects when assessing pain, we could not identify any instances where nurses documented external influences to scoring despite this being part of the NICU's mPAT protocol (e.g., noisy environment disturbing sleep pattern) [24]. Nevertheless, these possible explanations require further research to identify which specific descriptors (e.g., extended and clenched fists) nurses are recognizing more frequently across PMA groups.

### 4.3 Nurses' Perception of Pain

By analyzing the behavioral and physiological indicators on the mPAT separately, our results suggest that more infants expressed pain behaviorally. This analysis has provided further evidence that behavioral and physiological indicators of pain do not necessarily correlate with one another [9]. Nurses' perception of pain may enable a context‐informed evaluation of pain but should be used in combination with behavioral and physiological indicators. Future research should explore how nurses score this final criterion of the mPAT to further refine how the score can be utilized to improve the detection of pain and increase inter‐rater reliability.

### 4.4 Strengths and Limitations

This study had several strengths. Firstly, the study used a dataset that was acquired from a clinical cohort of interest. Secondly, this study was inclusive of infants with potential neurological impairments and did not exclude any gestational age groups. However, because this study was a retrospective record review, it was difficult to estimate the extent of missing data. This also meant we were unable to accurately identify the number of painful procedures infants underwent prior to the assessment of their pain. Finally, given the retrospective nature of this study, several unknown factors—for example, time of assessment and proximity to a painful procedure, and use of analgesia—may have influenced the findings which would better be controlled for in a prospective study. It should also be noted that there were approximately six times fewer infants in the youngest PMA group compared with the oldest PMA group. It is possible that clinicians in the unit may have administered the mPAT on younger infants only if they displayed highly visible signs of pain.

## 5 Conclusions

The assessment of nonacute pain in NICUs is a relatively underexplored area of research that is urgently needed to improve pain detection. This study evaluated the changes in total, behavioral, and physiological indicators of pain using the mPAT in relation to PMA at assessment and neurological impairment. Although this study demonstrated that postmenstrual age and neurological impairment may not affect the expression of pain as assessed by the mPAT, further work is needed to validate the mPAT as a measure of nonacute pain. Furthermore, the high behavioral responses in younger infants and neonates are unusual which requires further exploration in a future prospective study.

## Ethics Statement

This project (LNR/18/SCHN/298) was approved by the Human Research Ethics Committee of the Sydney Children's Hospital Network.

## Consent

This was a retrospective medical record review.

## Conflicts of Interest

The authors declare no conflicts of interest.

## Data Availability

Please make reasonable requests to the corresponding author for access to de‐identified data.
